# Choice of Donor Source and Conditioning Regimen for Hematopoietic Stem Cell Transplantation in Sickle Cell Disease

**DOI:** 10.3390/jcm8111997

**Published:** 2019-11-15

**Authors:** Emily Limerick, Courtney Fitzhugh

**Affiliations:** Cellular and Molecular Therapeutics Branch, National Heart, Lung, and Blood Institute, Bethesda, MD 20892, USA; emily.limerick@nih.gov

**Keywords:** sickle cell disease, allogeneic hematopoietic stem cell transplantation, human leukocyte antigen (HLA) matched sibling, haploidentical transplantation, alternative donor, graft-versus-host disease (GVHD)

## Abstract

In the United States, one out of every 500 African American children have sickle cell disease (SCD), and SCD affects approximately 100,000 Americans. Significant advances in the treatment of this monogenetic disorder have failed to substantially extend the life expectancy of adults with SCD over the past two decades. Hematopoietic stem cell transplantation (HSCT) remains the only curative option for patients with SCD. While human leukocyte antigen (HLA) matched sibling HSCT has been successful, its availability is extremely limited. This review summarizes various conditioning regimens that are currently available. We explore recent efforts to expand the availability of allogeneic HSCT, including matched unrelated, umbilical cord blood, and haploidentical stem cell sources. We consider the use of nonmyeloablative conditioning and haploidentical donor sources as emerging strategies to expand transplant availability, particularly for SCD patients with complications and comorbidities who can undergo neither matched related transplant nor myeloablative conditioning. Finally, we show that improved conditioning agents have improved success rates not only in the HLA-matched sibling setting but also alternative donor settings.

## 1. Introduction:

Sickle cell disease (SCD) was first described by James Herrick, MD, in 1910 [1]. He described the “peculiar elongated and sickle-shaped red blood corpuscles” that characterize this disorder. The missense mutation results from a single nucleotide substitution in the beta globin gene causing replacement of valine for glutamic acid in the codon for amino acid six. This results in hemoglobin polymerization upon deoxygenation and the transformation of red blood cells (RBC) from flexible biconcave discs to rigid, sickle-shaped RBCs which clog microvasculature. Patients, therefore, experience painful vaso-occlusive crises and organ damage including pulmonary hypertension, renal failure, liver disease, and heart failure.

In addition to both acute and chronic complications, SCD is associated with premature death. While there has been considerable improvement in median survival from 14.3 years in 1973 to about 45 years in 1994 (42 for males and 48 for females) [2], the median age at death for SCD has not changed since 1994. We recently reported a median age at death of 46 years for adults with SCD [3]. Thus far, hematopoietic stem cell transplantation (HSCT) remains the only curative option which could improve both quantity and quality of life. HSCT involves the infusion of stem cells from a selected donor, autologous (self) or allogeneic (different donor), and requires some degree of ablation of the recipient’s own hematopoiesis. While myeloablative conditioning (MAC) regimens cause irreversible cytopenia and require stem cell support, nonmyeloablative regimens involve less toxic conditioning and allow autologous stem cell recovery if the transplant fails. Reduced intensity regimens employ less chemotherapy and radiation and fall between myeloablative and nonmyeloablative in their strength and toxicity. Graft-versus-host disease (GVHD) can occur after allogeneic transplant when the donor’s immune cells attack the recipient’s host tissues. It is a serious, potentially lethal process and as such is a significant cause of morbidity and mortality post-transplant. While the risk varies with graft type and conditioning regimen, GVHD remains particularly problematic in the setting of sickle cell disease where there is no benefit from the graft-versus-host effect.

## 2. Myeloablative HLA-Matched Sibling HSCT Is Efficacious for Children with SCD

The first multicenter MAC human leukocyte antigen (HLA) matched sibling donor (MSD) HSCT report included 22 pediatric patients with symptomatic SCD and demonstrated that HSCT can cure SCD [4]. Two patients developed grade II or III acute graft-versus-host disease (aGVHD); the patient with grade III aGVHD subsequently died of chronic graft-versus-host disease (cGVHD). Of the surviving patients, 16 of 20 (80%) sustained engraftment. At four years of follow-up, disease free survival (DFS) was 73% and overall survival (OS) was 91%.

A more recent series of 87 pediatric patients examined results of related allogeneic HSCT after MAC in patients with a severe SCD phenotype [5]. They reported 93.1% and 86.1% OS and DFS, respectively, with a significant improvement in DFS to 95.3% in the second half of the study. The addition of anti-thymocyte globulin (ATG) to mitigate high observed rates of unstable mixed chimerism and subsequent graft rejection largely prevented graft rejection. A five-year cumulative incidence of rejection decreased from 22.6% to 2.9% with the addition of ATG. Furthermore, its use was associated with an increased frequency of mixed but stable chimerism. The principal complication was GVHD, which was responsible for four deaths. Twenty percent developed grade II or higher aGVHD, 8.1% of whom developed grade III to IV aGVHD. The cumulative incidence of cGVHD was 12.6%.

A 2017 survey included 1000 patients with SCD who underwent HLA-matched sibling HSCT between 1986 and 2013 and included data which were reported to international registries [6]. The results were notable for an excellent five-year OS of 92.9% and DFS of 91.4%. Age was an important factor associated with survival; five-year OS was 95% and DFS was 93% for patients younger than 16 years of age as compared with 81% for both OS and DFS among patients older than 16 years of age. This was not surprising since most patients received myeloablative conditioning. Furthermore, the time period of HSCT was associated with survival. Patients transplanted after 2006 had improved DFS. In this cohort, the cumulative incidence of grade II and IV aGVHD was 14.8% and cGVHD 14.3%. Myeloablative conditioning with busulfan, cyclophosphamide, and ATG has achieved the best HSCT outcomes in the MSD setting and is commonly employed in children and young adults [7]. Therefore, matched sibling HSCT with MAC has become a standard approach. While controversial, some recommend that patients with an MSD consider transplantation early in the disease course to optimize successful outcomes [8,9]. Fourteen pediatric SCD patients with asymptomatic disease received MSD transplants in Belgium to help pre-empt the need for medically complex and more frequent care prior to returning to their African country of origin. Overall and DFS were 100% and 93%, respectively, as compared with 88% and 80% in the cohort transplanted due to presence of severe disease; this study demonstrated the advantage of early intervention, before the occurrence of severe symptoms [9].

## 3. Reduced Intensity Conditioning Is Associated with Decreased Toxicity and Low Graft Failure

Matched sibling HSCT with MAC is associated with high cure rates and low rates of graft rejection but is associated with an increased risk of organ toxicity, as well as delayed sequelae such as infertility and secondary malignancy. Reduced-intensity conditioning (RIC) has been associated with stable donor engraftment [10]. In the largest series to date, of 43 SCD patients who received HSCT after a RIC of alemtuzumab, fludarabine, and melphalan, only the sole umbilical cord blood recipient experienced graft rejection, however, the risk of GVHD was significant with 23% developing aGVHD and 13% cGVHD. Others have reported promising results with RIC describing 100% OS and 86% to 100% DFS, as well as grade 2 to 4 aGVHD rates ranging from 0% to 17% and cGVHD from 0 to 11% [11,12,13]. In a cohort of pediatric patients who received RIC, long-term follow-up revealed normal growth velocities in 95% of the patients [14]. All post-pubertal females at time of transplantation resumed menstruation post-transplant and 67% (2/3) of these post-pubertal females had uneventful pregnancies during the follow-up period. There were no cases of late rejection in this survey. Additional long-term follow-up studies are indicated to evaluate whether toxicity is decreased with RIC as compared with MAC.

## 4. HLA-Matched Sibling HSCT Using Nonmyeloablative Conditioning Is Efficacious and Spares Morbidity

While RIC protocols may spare toxicity, nonmyeloablative approaches employ increased immunosuppression (vs. myelosuppression), and therefore may further reduce sequelae and provide a curative treatment option for patients with increased comorbidities. We recently reported 67 patients who underwent nonmyeloablative HSCT; we had serially measured sickle hemoglobin (HbS) and percent donor chimerism levels and followed their clinical manifestations. Three patients whose donors had sickle cell trait had high donor myeloid chimerism (DMC) initially, but then slowly falling levels over time [15]. As long as the mean DMC was at least 20%, the mean percentage of HbS remained below 50%, similar to their donors. Importantly, the patients remained free of SCD-related symptoms in this setting while a DMC <20% was associated with return of SCD. A greatly shortened RBC lifespan characterizes SCD. Our mathematical model suggested that because of the differences in RBC survival between donor and recipient cells, a minority of donor cells is adequate to reverse the sickling phenotype.

Novel strategies to promote tolerance and stable mixed chimerism, as well as decrease GVHD, which is particularly important in the nonmalignant setting where there is no “graft-versus-sickle” effect, are expanding. Sirolimus’ mammalian target of rapamycin (mTOR) mechanism promotes tolerance by blocking T cell proliferation without inhibiting T cell receptor-induced activation [16]. We previously employed a murine F1 into parent model where mice were conditioned with 300cGy total body irradiation (TBI) and received sirolimus or CSA for 30 days, after which immunosuppression was discontinued and chimerism levels monitored. This minimal conditioning regimen induced no significant morbidity and, unlike CSA, sirolimus was associated with stable mixed chimerism in the absence of immunosuppression. A nonmyeloablative conditioning regimen could facilitate allogeneic HSCT in adults with severe SCD, in whom the toxicity of MAC can be prohibitive. The challenges of nonmyeloablative conditioning include high rates of graft failure and GVHD due to alloreactive recipient or donor T cells, respectively. A cohort of 10 adults with a severe SCD phenotype received HLA-matched sibling peripheral blood stem cell (PBSC) transplantation after conditioning with 300cGy TBI and alemtuzumab; sirolimus was employed for GVHD prophylaxis [17]. Patients tolerated the conditioning well. Nine of 10 patients had stable engraftment with reversal of their SCD. Notably, there were no cases of acute or chronic GVHD or transplant-related mortality (TRM). The regimen was extended to 30 adults with similarly encouraging results: 87% had long-term stable donor engraftment without acute or chronic GVHD and fifteen engrafted patients discontinued immunosuppression with continued stable donor chimerism [18].

These results were duplicated at other institutions where DFS rates ranged from 91% to100% and no significant GVHD was reported. Thirteen high risk adults with SCD and a mean hematopoietic cell transplant comorbidity index of 3.3 underwent HSCT at the University of Chicago, Illinois [19]. All patients had initial engraftment and 92% maintained stable mixed chimerism. All patients were alive at study completion. Furthermore, a study including 51 SCD patients in Saudi Arabia compared 17 children who received MAC (composed of cyclophosphamide, busulfan, and ATG or thiotepa, busulfan, and fludarabine with CSA and methotrexate for GVHD prophylaxis) and 34 adults, who received the NIH nonmyeloablative preparative regimen [20]. There was no acute or chronic GVHD among the adult patients. Two pediatric patients had mild grade I aGVHD and no pediatric patients developed cGVHD. DFS was 90% in adult patients and 100% in children at two years post-transplant. OS at two years was 97% and 100% in adults and children, respectively. Similarly, the NIH regimen was employed in a population of 16 pediatric patients [21]. For the average 19.5-month follow-up period, there were no cases of graft rejection, aGVHD, or cGVHD. All patients were alive at the end of the follow-up period. Furthermore, they demonstrated sirolimus weaning was possible in the pediatric population by one year post-HSCT. All patients who were able to discontinue sirolimus maintained their graft off immunosuppression.

These data suggest that 300 cGy TBI creates adequate space in the bone marrow to establish sufficient donor hematopoiesis to reverse the SCD phenotype. Alemtuzumab’s lymphocytotoxic effects endure for weeks after administration, thereby decreasing recipient lymphocytes and depleting donor alloreactive T cells after transplantation. These effects serve to promote tolerance and prevent GVHD. In addition to the previously described role of sirolimus in tolerance induction, it has a role in decreasing GVHD risk by promoting differentiation of regulatory and helper T cells [22].

## 5. Alternative Donor Sources Improve Transplant Accessibility

While HLA-matched sibling HSCT has demonstrated efficacy, the vast majority of SCD patients do not have an HLA-matched sibling [23]. In a cohort of 287 patients who had HLA typing performed, 36% (*n* = 102) had a 6/6 HLA-matched sibling and 17% (*n* = 50) of these were eligible for transplant [18]. However, many of these patients were referred because they were already known to have an HLA-matched sibling. Instead, in the general community, <15% of patients with SCD are expected to have an HLA-matched sibling [23]. In a cohort of 113 children with SCD receiving chronic transfusion therapy, 40 (35%) had an unaffected full sibling and only eight (7%) identified an unaffected HLA-matched sibling [24]. Thus, alternative transplant sources are critical to expand accessibility.

### 5.1. Umbilical Cord Transplants

Cord blood (CB) is being used as an alternative source of hematopoietic cells for HSCT. Potential advantages of using hematopoietic stem cells from CB include a lower incidence of GVHD, its “off-the-shelf” availability, diminished risk of latent viral transmission, and negligible donor morbidity [31]. While CB grafts are reduced in cellularity and volume, their proliferative capacity and fraction of stem cells are often greater than other sources [32]. The use of CB transplantation is largely limited to the pediatric population secondary to the lower total cell dose.

#### 5.1.1. Related Umbilical Cord Transplants

Results from pediatric SCD patients who were treated by CB transplant (CBT) from a sibling donor and reported to the Eurocord registry revealed no TRM in two separate reports [26,29]. The Eurocord DFS rate improved from 67% in 1998 [26] to 90% in 2013 [29]. These improved results were found to be associated with an unfavorable impact of methotrexate (MTX) as GVHD prophylaxis, and therefore its use decreased with time. The data also suggest that the use of ATG and thiotepa may also be associated with improved outcomes in the CBT setting. The two-year probability of DFS is 90% for SCD patients, suggesting that related CBT is a safe curative strategy for hemoglobinopathies.

Stable engraftment is possible with the administration of single CB units, particularly when cell dose parameters are applied. However, neutrophil and platelet recovery are delayed as compared with bone marrow transplantation (BMT), contributing to higher TRM due to infection and hemorrhage during the prolonged period of pancytopenia that follows CBT. Overall median time to neutrophil engraftment was 25 days; platelet reconstitution occurred at a median of 45 days in the related CB transplantation setting (Table 1). Co-transplantation with HLA-identical sibling CB and BM demonstrated rapid neutrophil and platelet engraftment (17 and 29 days respectively), with no TRM, as well as lower GVHD rates [30]. Since 1996, 66 related CBT for SCD have been described in the literature. Overall, they are associated with 86% DFS, an 8% incidence of aGVHD, and no extensive cases of cGVHD. The OS was 89% (Table 1). Therefore, the efficacy of related CBT is comparable to other forms of HLA-matched sibling HSCT except with a lower incidence of extensive cGVHD as compared with BMT, however, as discussed above, the vast majority of patients do not have HLA-matched sibling donors.

#### 5.1.2. Unrelated Umbilical Cord Transplants

Cord blood transplants from MSD, therefore, do not expand the donor pool. Historically, the results for unrelated CBT for SCD have been disappointing with high rates of graft rejection. The sickle cell unrelated donor transplant (SCURT) trial included a cohort of eight patients who received unrelated CBT. The conditioning regimen consisted of alemtuzumab, fludarabine, and melphalan with CSA or mycophenolate mofetil (MMF) and tacrolimus for GVHD prophylaxis. The OS was 88% but the DFS was only 38% and five of eight patients had graft rejection and autologous blood count recovery. Notably, the median post-thaw total nucleated cell dose was 4.5 × 10^7^/kg with a range of 2.1–6.3 [33]. Ruggeri et al. report on CBT recommended that only units containing an expected infusion cell dose >5 × 10^7^/kg should be considered [34]. Enrolment into the CB cohort of the SCURT trial was prematurely suspended due to high rates of graft rejection.

More recent data suggest that the addition of thiotepa to this RIC regimen could improve outcomes in the setting of unrelated umbilical CBT. Abraham and colleagues achieved 100% and 78% one-year OS and DFS, respectively. Their cohort experienced 33% aGVHD (grade II to IV), 22% mild cGVHD, and 11% chronic extensive GVHD [35]. The median total nucleated cell dose was 5.9 × 10^7^/kg (range, 3.9 to 8.5). Toxicity and prolonged cytopenias remain a challenge for CBT from unrelated donors. Overall median time to neutrophil engraftment was 24 days; platelet reconstitution occurred at a median of 50 days for unrelated CB recipients (Table 2). Table 2 summarizes the pediatric unrelated CBT experience for SCD. Thus, while unrelated CBT has the potential to extend the donor pool, particularly in the pediatric population, DFS is lower as compared with the HLA-matched sibling setting, and there is a higher risk of GVHD.

### 5.2. Matched Unrelated Donor Transplant

Matched unrelated donor (MUD) HSCT, where unrelated donor registries provide potential donor sources, is a viable alternative for patients who do not have an HLA-matched sibling. A National Marrow Donor Program search reported the highest probability of finding an optimal donor among whites of European descent (75%); black Americans of all ethnic backgrounds had among the lowest probability (16% to 19%) [39]. As most SCD patients in the United States are black Americans, this donor source is also limited.

The MUD arm of SCURT was a multicenter phase II trial whose aim was to utilize HLA-matched unrelated donors to expand the donor pool [40]. The trial included 29 children with a median age of 14 years. The preparative regimen included alemtuzumab, fludarabine, and melphalan. They report 86% one-year overall survival and 76% overall survival at the time of publication. Six patients died of GVHD and one patient died following a second transplant. The one-year DFS rate was 75%. The regimen was associated with 28% aGVHD (grade II to IV) and 38% chronic extensive GVHD. The timing of alemtuzumab administration three weeks before graft infusion may have contributed to this higher rate of GVHD. This early administration was designed to overcome host rejection of the graft by preventing waning alemtuzumab levels by the time of donor cell infusion, and thereby optimize donor T cell engraftment.

A reduced toxicity preparative regimen of busulfan, fludarabine, and ATG was employed in five adolescent and young adult patients receiving MUD BMT. Four of five patients were alive and tree were free of SCD; a fourth patient was free of SCD after a second transplant. One patient developed grade 3 aGVHD and one developed severe cGVHD [41]. These pilot trial results support a multicenter phase II clinical trial (BMT CTN #1503) testing this reduced-toxicity conditioning regimen in both HLA-MSD and HLA-MUD BMT in adults with SCD. This clinical trial (NCT02766465) is currently recruiting and will compare BMT to standard of care in individuals without a suitably HLA-matched related or MUD. Table 3 summarizes the matched unrelated donor transplants for SCD published to date. Therefore, while overall the efficacy of MUD transplants is higher than unrelated CBT, it remains lower than MSD HSCT, and GVHD remains a significant challenge, especially in the pediatric setting. Furthermore, the majority of patients with SCD will not have a MUD available.

### 5.3. Haploidentical HSCT 

Haploidentical donors are the most accessible donor population; almost everyone will have parents, children, and half-matched siblings who can serve as donors. Haploidentical HSCT affords the possibility of large cell doses, as well as the potential for repeat donor collections. Early studies demonstrated its potential to halt the progression of end organ damage: one long-term follow-up reported no patient with sustained engraftment exhibited any clinical signs of stroke or progression of prior cerebrovascular accident on imaging studies, no patient experienced change in cardiac shortening fraction, and all patients with avascular necrosis had stabilization of disease [45]. The significant risks remain graft rejection and lethal GVHD.

Numerous strategies have been employed to decrease the GVHD risk, including donor T-cell depletion and tolerance induction in host-reactive donor T cells [46]. A murine model demonstrated that durable engraftment in the haploidentical setting is possible after nonmyeloablative conditioning with fludarabine, low-dose TBI, and post-transplant cyclophosphamide (PT-Cy) [46]. PT-Cy has been broadly implemented as a GVHD prophylaxis strategy. It prevents severe acute and chronic GVHD, while preserving infectious immunity, ultimately resulting in low rates of nonrelapse mortality [47]. Moreover, PT-Cy facilitates in vivo alloreactive functional T-cell depletion and mediates tolerance induction. This is a multistep process characterized by” clonal destruction” of proliferating allo-stimulated T cells, peripheral tolerance development, and thymic deletion of donor progenitor cell-derived alloreactive T cells [48].

PT-Cy has also proven efficacious in children and young adults with high risk hematologic malignancies receiving nonmyeloablative haploidentical HSCT. Forty patients ages one to 25 years received haploidentical transplants with GVHD prophylaxis consisting of PT-Cy, MMF, and tacrolimus [49]. At six months post-transplant, the cumulative incidence of grade III to IV aGVHD was 13% while the cumulative incidence of moderate-severe cGVHD at two years was 7%. The rate of engraftment was 94%.

Several putative mechanisms for PT-Cy’s role in GVHD prevention have been suggested. Earlier work has suggested that although proliferating lymphocytes are depleted, there is relative resistance of T-regulatory cells to PT-Cy, which then mediate the anti-GVHD effects [50]. However, a more recent study was performed in a haploidentical murine model designed to test the hypothesis that elimination of alloreactive T cells is a mechanism by which PT-Cy prevents GVHD after HSCT. The results suggested that alloreactive T cells become functionally impaired, but not depleted, after PT-Cy [51]. Furthermore, there is preferential expansion of T-regulatory cells, which play an increasingly important role with time. While the definitive mechanism of PT-Cy in GVHD prevention has yet to be fully elucidated, one theory is that PT-Cy induces alloreactive T cell exhaustion, which facilitates its anti-GVHD effects.

The role of PT-Cy in the haploidentical setting was further explored in a cohort of 14 patients with severe hemoglobinopathies [52]. The patients received a preparative regimen of ATG, fludarabine, cyclophosphamide, and low-dose TBI, as well as GVHD prophylaxis consisting of PT-Cy, MMF, and tacrolimus or sirolimus, which demonstrated efficacy at preventing GVHD. Forty-three percent of haploidentical pairs experienced graft rejection, and an additional patient had insufficient donor chimerism levels to maintain donor-type hemoglobin, as the patient had severe anemia and a sickle hemoglobin level much higher than the donor. Of the 11 with durable engraftment, 45% (*n* = 5) had mixed chimerism and 75% of engrafted patients were able to wean off immunosuppressive therapy. None of the patients who received haploidentical grafts developed GVHD. This work reinforces the paradigm that limiting GVHD is usually associated with increasing mixed chimerism [52]. Achieving tolerance induction and stable mixed chimerism is a novel strategy which balances these competing points and research in this area is ongoing.

Co-stimulation blockade (COSBL) with CTLA4 immunoglobulin, for example abatacept, induces transplant tolerance and has been effectively employed as both GVHD prophylaxis and treatment [63]. Indeed, in pediatric patients with severe aplastic anemia (SAA), the rates of survival free of GVHD and SAA at one year were significantly higher in the COSBL group (80% vs. 30% in the control group). Preliminary findings suggest that COSBL has a synergistic effect with PT-Cy and sirolimus. Employing abatacept as a novel agent to prevent GVHD in the HSCT for SCD is another strategy currently being explored.

We wanted to develop a regimen that was built from the success seen in the HLA-matched sibling setting with nonmyeloablative conditioning. We developed a regimen consisting of dose escalation of PT-Cy based on the results seen in our murine model where PT-Cy and sirolimus were synergistic in the lymphocyte replete setting, but in the lymphocyte deplete setting, PT-Cy did not improve donor chimerism levels [64]. We transplanted 21 SCD and two transfusion-dependent beta thalassemia patients who received PBSCs from haploidentical donors after nonmyeloablative conditioning with alemtuzumab, TBI (400 cGy total), sirolimus, and escalating doses of PT-Cy (0 to 100 mg/kg total) [53]. This study included patients with significant disease complications including cirrhosis, dialysis, and pulmonary hypertension and the mean HCT comorbidity index was 6 ± 2.3. Despite the severe organ damage, patients tolerated the conditioning regimen with no patients dying prior to day 100. Post-transplant cyclophosphamide improved donor engraftment, with 83% engraftment at the 100mg/kg dose as compared with 33% in the no PT-Cy cohort. While there was no significant GVHD, this was at the cost of high graft rejection; from 100% (0/3) in those who received no PT-Cy to 50% (6/12) in the highest PT-Cy dose group [53].

Additional up-front conditioning and T-cell depletion have improved the outcomes for haploidentical HSCT. A modified Hopkins regimen with 300 cGy (instead of 200 cGy) TBI and PBSC (in lieu of bone marrow) was employed in a cohort of eight SCD patients. The disease-free and overall survival were 75% and 87.5%, respectively. Two patients developed aGVHD and only one patient experienced cGVHD [57]. Another modified Hopkins regimen, this with the addition of thiotepa, was given to 15 SCD patients. The disease-free and overall survival were 93% and 100%, respectively after a median follow-up of 13 months. While aGVHD was substantial with 33% developing aGVHD, 13% were grade 3 to 4, and there was only one occurrence of moderate cGVHD (7%) [60]. BMT CTN 1507 (NCT03263559) will expand on this study with a phase II multicenter trial to evaluate the efficacy and toxicity of haploidentical BMT in children and adults with SCD after preconditioning with HU and conditioning with a regimen of ATG/fludarabine/cyclophosphamide/200cGy TBI and thiotepa. Children must have a history of stroke and adults must meet eligibility criteria for severe SCD.

Efforts at ex vivo T-cell depletion have employed CD3/CD19 or TCRαβ/CD19 reduction strategies. In a study of predominantly pediatric SCD patients (ages four to 31, median = 15) receiving CD3/CD19- or TCRαβ/CD19-depleted grafts, all patients had primary engraftment [62]. Although patients >18 years of age had OS and DFS of 100%, there was 10% overall mortality (two patients died from macrophage activation syndrome and CMV pneumonitis). Furthermore, one patient developed severe BK virus nephritis and required renal replacement therapy.

Numerous transplant regimens and graft types have been employed. When combining all of the studies reported since 2017, the DFS for haploidentical transplant is 86%, while the overall survival is 93%. The DFS and OS from studies since 2018 alone improve to 91% and 94%, success rates which compare favorably with the HLA-matched sibling HSCT setting. These data suggest that haploidentical HSCT may offer a widely available curative strategy which is accessible even to patients with severe SCD manifestations. Table 4 summarizes the results of recent studies of haploidentical HSCT for SCD.

## 6. Comparative HSCT Outcomes by Donor Type

**Figure 1:**
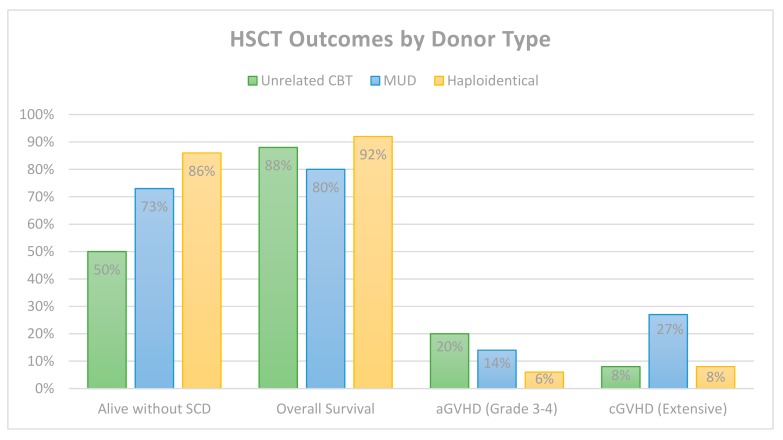
Comparative HSCT outcomes. *n* = 50 for unrelated CBT, *n* = 44 for MUD and *n* = 100 for haploidentical. HSCT, hematopoietic stem cell transplant; SCD, sickle cell disease; aGVHD, acute graft versus host disease; cGHVD, chronic graft versus host disease; CBT, cord blood transplant; and MUD, matched unrelated donor.

There are limited data comparing transplant outcomes for SCD by donor type. Our previous review reported the highest DFS (75%) in the MUD setting as compared with haploidentical (64%) and unrelated CBT (48%) [65]. A 2019 retrospective cohort study of 90 US centers evaluated data reported to the Center for International Blood and Marrow Transplantation Research (CIBMTR) including allogeneic HSCT for sickle cell disease and sickle beta thalassemia [66]. Their data support previous reports that DFS is improved in younger patients. Furthermore, they report decreased DFS with RIC as compared with nonmyeloablative and after transplantation of non-HLA-matched sibling grafts. Whereas HLA-matched sibling transplant remains superior to alternative donor transplant, the data did not favor one alternative donor type over another. While clinical trials for the various alternative donor strategies improve efficacy while decreasing toxicity, the overall DFS of 86% and OS of 92% described in Table 4 indicate that currently haploidentical HSCT may be the preferred strategy in the absence of a matched sibling. Figure 1 provides a graphical summary of the comparative outcomes of the studies outlined in these pages, organized by donor type.

## 7. Conclusions

With overall and DFS in excess of 90%, HLA-matched sibling HSCT is increasingly becoming the standard of care for patients with severe disease with evidence demonstrating that younger patients have improved DFS. In the HLA-matched sibling setting, myeloablative conditioning, including ATG to mitigate graft rejection, has high efficacy, although GVHD remains a significant source of morbidity. Nonmyeloablative conditioning aimed at tolerance induction spares toxicity and studies thus far have not reported GVHD in adults or children, despite the use of PBSCs. The availability of an HLA-MSD is limited, with as few as 7% of SCD patients identifying an unaffected matched sibling in one cohort [24]. Therefore, expanding alternative donor options is critical to achieve transplant accessibility. With the elimination of MTX from GVHD regimens and the addition of ATG and thiotepa, excellent DFS rates have been reported with related CBT. However, related CBT have largely used 6/6 HLA-matched sibling donors, and therefore it has not helped expand the donor pool. While previous results of unrelated CBT have been disappointing, more recent data suggest that thiotepa, when added to a RIC regimen, may ameliorate outcomes [35]. Early alemtuzumab is associated with a high rate of GVHD for MUD transplants. Studies are ongoing to evaluate whether abatacept, with or without PT-Cy, may decrease the incidence of GVHD using a similar regimen (NCT03128996 and NCT02867800). A study is currently ongoing to compare a busulfan-based RIC regimen in MSD and MUD BMT to the standard of care (NCT 02766465). For unrelated umbilical cord and haploidentical HSCT, more intensive conditioning and T-cell depletion have achieved notable successes with a decreased graft rejection rate and lower incidence of GVHD, especially in the haploidentical setting. Furthermore, haploidentical donors are the most widely available allogeneic donor source. Indeed, results from the most recent studies demonstrate excellent outcomes that rival the HLA-matched sibling setting but with a much larger available donor pool. Longer follow-up is necessary to evaluate efficacy and to monitor for late effects of HSCT. Lastly, enrolment of patients in clinical trials should be encouraged with the goal of continuing to improve efficacy and decrease toxicity for patients with SCD undergoing HSCT. 

## Figures and Tables

**Table 1:** Pediatric related umbilical cord transplants for sickle cell disease (SCD).

Reference	Transplant Regimen	Number of Patients (Median Age)	Alive without SCD (%)	Median Time to Neutrophil/Platelet Recovery (Days)	Grade 3–4 Acute GvHD (% of Total)	Extensive Chronic (% of Total)	Mortality (Cause)
**Brichard, 1996 [25]**	Bu 16 mg/kg, Cy 200 mg/kg, ATG, CSA	1 (5)	1 (100%)	32/48	0	0	0
**Miniero, 1998 [26]**	Bu 16 mg/kg, Cy 200 mg/kg, CSA ± MTX	3 (3–11)	2 (67%)	31/40	0	0	0
**Gore, 2000 [27]**	Bu 726 mg/m^2^, Cy 200 mg/kg, ATG, CSA	1 (9)	1 (100%)	23/49	0	0	0
**Walters, 2005 ^†^ [28]**	NR	8 (NR)	6 (75%)	23/45	NR	NR	1 (intractable seizures)
**Matthes-Martin, 2013 [12]**	TBI (2 Gy), Flu 160 mg/m^2^, Mel 140 mg/m^2^, Alem 1mg/kg, CSA, MMF	1 (11.1)	1 (100%)	19/NR	0	0	0
**Locatelli, 2013 [29]**	Bu ± Flu ± Cy ± ATG/ALG ± TT, CSA ± MTX	30 (5.9)	27 (90%)	23/38	11 (11%) (Grade 2–3 only) *	0	3 (2 hemorrhage, 1 organ failure) *
**Soni, 2014 [30]**	Bu 14–16 mg/kg, Cy 200 mg/kg, ATG, CSA ± MMF, ± MTX, ± Pred	22 (5.2) *	19*	25/48	1*	0	3 (infection, pulmonary complications, seizure) *
**Total**	--	66	57 (86%)	25/45	12 (18%) *	0	7 (11%) *

Alem, alemtuzumab; ALG, anti-lymphocyte globulin; ATG, antithymocyte globulin; Bu, busulfan; CSA, cyclosporine; Cy, cyclophosphamide; Flu, fludarabine; GvHD, graft-versus-host disease; Mel, melphalan; MMF, mycophenolate mofetil; MTX, methotrexate; NR, not reported; Pred, prednisone; SCD, sickle cell disease; TBI, total body irradiation; TT, thiotepa; Treo, treosulfan. ^†^ included 4/6 HLA match patients (*n* = 4). * Includes patients with sickle cell disease and thalassemia.

**Table 2:** Pediatric unrelated umbilical cord transplants for SCD.

Reference	Transplant Regimen	HLA Match (*n*)	Number of Patients (Median Age)	Alive without SCD (%)	Median Time to Neutrophil/Platelet Recovery (Days)	Grade 3–4 Acute GvHD (% of Total)	Extensive Chronic GvHD (% of Total)	Mortality (Cause)
**Adamkiewicz, 2007 [36]**	Mixed, 4 pts myeloablative, 3 pts reduced intensity	5/6 (2) 4/6 (5)	7 (2.4)	3 (43%)	22/70	2 (28%)	1 (14%)	1 (multi-organ failure)
**Ruggeri, 2011 [34]**	Mixed, 9 pts myeloablative, 7 pts reduced intensity	6/6 (2) 5/6 (4) 4/6 (10)	16 (6)	8 (50%)	22/40	3 (19%) *	2 (12%) *	1 (aGvHD)
**Kamani, 2012 [33]**	Alem 48 mg, Flu 150 mg/m^2^, Mel 140 mg/m^2^, CSA or Tac + MMF	6/6 (1) 5/6 (7)	8 (13.7)	3 (38%)	22/41	0	1 (12%)	1 (cGvHD)
**Radhakrishnan, 2013 [37]**	Bu 12.8–16 mg/kg, Flu 180 mg/m^2^, Alem 54 mg, MMF, Tac	NR	8 (3.6)	4 (50%)	34/54	2 (25%)	0	3 (infection)
**Kharbanda, 2014 [38]**	Flu 150 mg/m^2^, Mel 140 mg/m^2^, Alem 60 mg, CSA, MMF	4/6 (2)	2 (8)	0	24/46	0	0	0
**Abraham, 2017 [35]**	Alem, Flu 30 mg/m^2^, TT 4 mg/kg, Mel 140 mg/m^2^, CSA or Tac	6/6 (2) 5/6 (7)	9 (4)	7 (78%)	21/52	3 (43%)	0	0
**Total**	--	6/6 (5) 5/6 (20) 4/6 (17)	50	25 (50%)	24/50	10 (20%)	4 (8%)	6 (12%)

Alem, alemtuzumab; Bu, busulfan; CSA, cyclosporine; Flu, fludarabine; GVHD, graft-versus-host disease; HLA, human leukocyte antigen; Mel, melphalan; MMF, mycophenolate mofetil; NR, not reported; pts, patients; SCD, sickle cell disease; Tac, tacrolimus; TT, thiotepa. * Includes patients with sickle cell disease and thalassemia.

**Table 3:** Matched unrelated donor transplants for SCD.

Reference	Transplant Regimen	Graft Type	Number of Patients (Median Age)	Alive without SCD (%)	Median Time to Neutrophil/Platelet Recovery (Days)	Grade 3–4 Acute GvHD (% of Total)	Extensive Chronic GvHD (% of Total)	Mortality (Cause)
**Strocchio, 2015 [42]**	TT 10 mg/kg, Treo 42 g/m^2^, Flu 160 mg/m^2^, ATG 15–30 mg/kg, CSA, MTX	BM (5) PBSCs (1)	6 (8.4)	5 (83%)	21/26	0	0	0
**Shenoy, 2016 [40]**	Alem 45 mg, Flu 150 mg/m^2^, Mel 140 mg/m^2^, CSA or Tac, MTX, Methylpred	BM	29 (14)	20 (69%)	12/24	5 (17%)	11 (38%)	7 (GVHD) 1 (following 2nd transplant)
**Marzollo, 2017 [43]**	TT 8–10 mg/kg, Treo 42 g/m^2^, Flu 160 mg/m^2^, ATG 60 mg/kg, ± MTX, CSA	BM or T-cell depleted PBSCs *	2 (8.5)	2 (100%)	20/22	0	0	0
**Gilman, 2017 [44]**	Mel 140 mg/m^2^, TT 10mg/kg, Flu 200 mg/m^2^, ATG 10 mg/kg ± Ritux 150-375 mg/m^2^, MTX	PBSCs	2 (9)	2 (100%)	14/19	0	0	0
**Krishnamur-ti, 2019 [41]**	Bu 13.2 mg/kg, Flu 175 mg/m^2^, ATG, CSA or Tac, MTX	BM	5 (22.5)	3 (60%)	17/21	1 (20%)	1 (20%)	1 (cGvHD)
**Total**	Mixed	BM or PBSCs	44	32 (73%)	17/22	6 (14%)	12 (27%)	9 (20%)

Alem, alemtuzumab; ATG, anti-thymocyte globulin; BM, bone marrow; Bu, busulfan; CSA, cyclosporine; Flu, fludarabine; GVHD, graft-versus-host disease; Mel, melphalan; Methylpred, methylprednisolone; MTX, methotrexate; PBSC, peripheral blood stem cells; SCD, sickle cell disease; Ritux, rituximab; Tac, tacrolimus; Treo, treosulfan; TT, thiotepa. * T-cell depleted PBSCs were infused for the 1 mismatched unrelated donor transplant.

**Table 4:** Haploidentical hematopoietic stem cell transplantation (HSCT) for SCD.

Reference	Transplant Regimen	Graft Type	Number of Patients (Median Age)	Alive without SCD (%)	Median Time to Neutrophil/Platelet Recovery (Days)	Grade 3–4 Acute GvHD (% of Total)	Extensive Chronic GvHD (% of Total)	Mortality (Cause)
**Fitzhugh, 2017 [53]**	Alem 1mg/kg, 400 cGy TBI, PT-Cy 100 mg/kg, Sir	PBSC	12 (36)	6 (50%)	27/31	0	0	1 (PH and CHF)
**Foell, 2017 [54]**	TT 10 mg/kg, Flu 160 mg/m^2^, Treo 42 g/m^2^, ATG 45 mg/kg, CSA, MMF	CD3/ CD19 depleted PBSC	9 (16)	8 (89%)	18/11	0	1 (moderate) (12.5%)	1 (CMV)
**Marzollo, 2017 [43]**	TT 8–10 mg/kg, Treo 42 g/m^2^, Flu 160 mg/m^2^, ATG 20 mg/kg ± Ritux 200 mg/m^2^	TCRab/ CD19 depleted PBSC	2 (15)	2 (100%)	20/22	0	0	0
**Wiebking, 2017 [55]**	Alem 0.4 mg/kg, Flu 150 mg/m^2^, Treo 42 g/m^2^, TT 10 mg/kg, PT-Cy 100 mg/kg, MMF, Tac	BM	3 (8)	3 (100%)	17/25	0	0	0
**Gilman, 2017 [44]**	Mel 140 mg/m^2^, TT 10 mg/kg, Flu 200 mg/m^2^, ATG 10 mg/kg ± Ritux 375 mg/m^2^, MTX	PBSC	8 (16)	7 (88%)	14/19	1 (12.5%)	1 (12.5%)	1 (GvHD)
**Frangoul, 2018 [56]**	Cy 29 mg/kg, Flu 150 mg/m^2^, 200 cGy TBI, ATG 4.5 mg/kg, TT 10 mg/kg, PT-Cy 100 mg/kg, Sir + MMF	Primed BM or PBSC	4 (14.3)	4 (100%)	22/26	0	0	0
**Saraf, 2018 [57]**	Cy 29 mg/kg, Flu 150 mg/m^2^, 300 cGy TBI, ATG 4.5 mg/kg, PT-Cy 100 mg/kg, Tac or Sir + MMF	PBSC	8 (28)	6 (75%)	22/NR	1 (12.5%)	1 (moderate) (12.5%)	1 (unknown)
**Pawlowska, 2018 [58]**	Flu/Dex Pre-Conditioning, ATG 4.5 mg/kg, Flu 210 mg/m^2^, Bu 520 mg/m^2^, PT-Cy 100 mg/kg, Tac, MMF	BM or PBSC	4 (19)	4 (100%)	16/19	0	0	0
**Gaziev, 2018 [59]**	HU/Aza/Flu Pre-Conditioning, Bu 14 mg/kg, TT 10 mg/kg, Cy 200 mg/kg, ATG 12.5 mg/kg, CSA, MMF or methylpred	TCRαβ/CD19 depleted PBSCs	3 (7)	3 (100%)	13/15	1 (33%) *	3 (21%) *	2 *
**de la Fuente, 2019 [60]**	Cy 29 mg/kg, Flu 150 mg/m^2^, 200 cGy TBI, ATG 4.5 mg/kg, TT 10 mg/kg PT-Cy 100 mg/kg, Sir + MMF	Primed BM	15 (20.4)	14 (93%)	22/28	2 (13%)	1 (moderate) (6.7%)	0
**Bolaños-Meade, 2019 [61]**	ATG, Flu 30 mg/m^2^, Cy 14.5 mg/kg, TBI 400cGy, PT-Cy 100 mg/kg, MMF, Sir	BM	12 (16)	11 (92%)	28/26	1 (8.3%) *	1 (moderate) (8.3%) *	0
**Foell, 2019 [62]**	TT 10 mg/kg, Flu 160 mg/m^2^, Treo 42 g/m^2^, ATG 45 mg/kg, CSA, MMF	CD3/CD19- or TCRαβ/CD19-depleted PBSC	20 (14.5)	18 (90%)	19/10	0	0	2 (CMV, MAS)
**Total**	Mixed	Mixed	100	86 (86%)	20/31	6 (6%)	8 (8%)	8 (8%)

Alem, alemtuzumab; ATG, anti-thymocyte globulin; Aza, azacytidine; BM, bone marrow; Bu, busulfan; CD, cluster of differentiation; cGy, centigray; CHF, congestive heart failure; CMV, cytomegalovirus; CSA, cyclosporine; Cy, cyclophosphamide; Dex, dexamethasone; Flu, fludarabine; GvHD, graft-versus-host disease; HU—hydroxyurea; MAS, macrophage activation syndrome; Mel, melphalan; methylpred, methylprednisolone; MMF, mycophenolate mofetil; MTX, methotrexate; PBSC, peripheral blood stem cell; PH, pulmonary hypertension; PT-Cy, post-transplant cyclophosphamide; Ritux, rituximab; SCD, sickle cell disease; Sir, sirolimus; Tac, tacrolimus; TBI, total body irradiation; TCR, T cell receptor; Treo, treosulfan; TT, thiotepa. * Includes patients with sickle cell disease and thalassemia.
